# Genetic Variation and Reproductive Timing: African American Women from the Population Architecture Using Genomics and Epidemiology (PAGE) Study

**DOI:** 10.1371/journal.pone.0055258

**Published:** 2013-02-12

**Authors:** Kylee L. Spencer, Jennifer Malinowski, Cara L. Carty, Nora Franceschini, Lindsay Fernández-Rhodes, Alicia Young, Iona Cheng, Marylyn D. Ritchie, Christopher A. Haiman, Lynne Wilkens, ChunyuanWu, Tara C. Matise, Christopher S. Carlson, Kathleen Brennan, Amy Park, Aleksandar Rajkovic, Lucia A. Hindorff, Steven Buyske, Dana C. Crawford

**Affiliations:** 1 Center for Human Genetics Research, Department of Molecular Physiology and Biophysics, Vanderbilt University, Nashville, Tennessee, United States of America; 2 Department of Genetics, Rutgers University, Piscataway, New Jersey, United States of America; 3 Department of Statistics, Rutgers University, Piscataway, New Jersey, United States of America; 4 Department of Epidemiology, University of North Carolina, Chapel Hill, North Carolina, United States of America; 5 Public Health Sciences, Fred Hutchinson Cancer Research Center, Seattle, Washington, United States of America; 6 Keck School of Medicine, University of Southern California, Los Angeles, California, United States of America; 7 University of Hawaii Cancer Center, University of Hawaii, Honolulu, Hawaii, United States of America; 8 Department of Obstetrics and Gynecology, University of California Los Angeles, Los Angeles, California, United States of America; 9 Department of Obstetrics and Gynecology, School of Medicine, Georgetown University, Washington, DC, United States of America; 10 Magee-Womens Research Institute, Department of Obstetrics, Gynecology and Reproductive Sciences, University of Pittsburgh, Pittsburgh, Pennsylvania, United States of America; 11 Office of Population Genomics, National Human Genome Research Institute, National Institutes of Health, Bethesda, Maryland, United States of America; 12 Department of Biology and Environmental Science, Heidelberg University, Tiffin, Ohio, United States of America; 13 Biochemistry and Molecular Biology, Penn State University, University Park, Maryland, United States of America; Peninsula College of Medicine and Dentistry, University of Exeter, United Kingdom

## Abstract

Age at menarche (AM) and age at natural menopause (ANM) define the boundaries of the reproductive lifespan in women. Their timing is associated with various diseases, including cancer and cardiovascular disease. Genome-wide association studies have identified several genetic variants associated with either AM or ANM in populations of largely European or Asian descent women. The extent to which these associations generalize to diverse populations remains unknown. Therefore, we sought to replicate previously reported AM and ANM findings and to identify novel AM and ANM variants using the Metabochip (n = 161,098 SNPs) in 4,159 and 1,860 African American women, respectively, in the Women’s Health Initiative (WHI) and Atherosclerosis Risk in Communities (ARIC) studies, as part of the Population Architecture using Genomics and Epidemiology (PAGE) Study. We replicated or generalized one previously identified variant for AM, rs1361108/*CENPW*, and two variants for ANM, rs897798/*BRSK1* and rs769450/*APOE*, to our African American cohort. Overall, generalization of the majority of previously-identified variants for AM and ANM, including *LIN28B* and *MCM8*, was not observed in this African American sample. We identified three novel loci associated with ANM that reached significance after multiple testing correction (*LDLR* rs189596789, p = 5×10^−08^; *KCNQ1* rs79972789, p = 1.9×10^−07^; *COL4A3BP* rs181686584, p = 2.9×10^−07^). Our most significant AM association was upstream of *RSF1*, a gene implicated in ovarian and breast cancers (rs11604207, p = 1.6×10^−06^). While most associations were identified in either AM or ANM, we did identify genes suggestively associated with both: *PHACTR1* and *ARHGAP42*. The lack of generalization coupled with the potentially novel associations identified here emphasize the need for additional genetic discovery efforts for AM and ANM in diverse populations.

## Introduction

Age at menarche (AM) and age at natural menopause (ANM) are components of the reproductive lifespan in women. Timing of these reproductive milestones is associated with various diseases and cancers such as type 2 diabetes, cardiovascular disease, endometrial and breast cancers, as well as with fertility issues [1]–[9].

Both cross-sectional and longitudinal studies have shown an overall decline in age of menarche in US girls from the 1960s to the 1990s [10]–[16]. These studies have also shown clear differences in age of sexual maturation of European Americans compared to African Americans, with African American girls attaining menarche earlier than European American girls [13]. Childhood obesity, higher in African American adolescents than other groups, has been linked to the earlier timing of menarche observed compared with European Americans [13], [17]–[19]. A genetic component for the timing of menarche has been investigated in numerous twin and large population studies, with heritability estimates ranging from 0.49 in the Fels Longitudinal Study to 0.72 in the Breakthrough Generations Study [11], [20]–[22].

Similar to the timing of age at menarche, the age at which natural menopause occurs is affected by multiple factors [23]. Active smoking is consistently associated with earlier menopause; however, the effects of exposure to other carcinogens and endocrine disruptors have not been completely elucidated [24]–[26]. Diet and obesity are also suggested to impact the timing of natural menopause [27], [28]. Based on twin studies and mother-daughter pairs, the heritability of age at natural menopause has been estimated to be between 44–63% [29]–[31]. Family history of the timing of these reproductive events is a strong predictor of both AM and ANM [29]–[31].

Genetic and environmental factors that determine AM and ANM have been considered in numerous studies, but many of these studies have conflicting or unreplicated results [32]–[34]. Furthermore, the majority of these studies have been performed in cohorts of largely European or Asian descent [35]–[39]. As a result, generalization of genetic associations with AM/ANM to other race/ethnicities is lacking. A recent review has noted the absence of studies with non-European-descent ethnicities and suggests expanding future studies to include other race/ethnicities [40] to identify genetic factors that influence AM/ANM across all populations. Replication of known ANM loci identified in European-descent women has been demonstrated in Hispanic women in the Women’s Health Initiative (WHI); however, to our knowledge, there has been no genome-wide association study (GWAS) or generalization study published to date on AM or ANM with an African American cohort [41].

In this study, we used data from the Metabochip genotyping array to characterize previously identified variants associated with menarche and menopause in African Americans in a combined cohort of African-American women from the Women’s Health Initiative (WHI) and Atherosclerosis Risk in Communities (ARIC) studies [42] as part of the Population Architecture using Genomics and Epidemiology (PAGE) Study [43]. The Metabochip array is based on the Illumina iSelect platform and contains approximately 200,000 single nucleotide polymorphisms (SNPs) consisting of GWAS index variants and fine-mapping common and less common variants for GWAS-identified regions relevant to metabolic and cardiovascular traits [43], [44]. Using current GWAS and candidate gene literature as a guide, we attempted to generalize previously identified menarche and menopause SNPs and gene regions identified in European-descent populations to African Americans in the PAGE Study. We then sought to identify novel SNPs associated with AM and/or ANM.

## Materials and Methods

### Study Participants

Women participants from two cohorts of the PAGE Study [42], Atherosclerosis Risk in Communities Study (ARIC) and the Women’s Health Initiative (WHI), were included in these analyses. ARIC is a population-based prospective study of cardiovascular diseases and their causes in ∼16,000 men and women aged 45–64 at baseline [45]. Participants were recruited in Forsyth County, N.C., Jackson, M.S., Minneapolis, M.N., and Washington County, M.D. From this group, 2,070 women, all of self-reported African American ancestry and with information on reproductive timing, were selected for study. The WHI is a long term national health study investigating the leading causes of mortality and frailty in post-menopausal women in the United States, including heart disease, breast and colorectal cancer, and osteoporotic fractures [46]. A subset of 2,455 self-reported African American women selected based on consent to use DNA and availability of DNA, blood lipids, and glucose and insulin measurements were included in this study. The appropriate institutional review board at each participating study site approved all procedures, and written informed consent was obtained from all participants.

### Definition of Age at Menarche and Age at Natural Menopause

Age at menarche was defined as the age when menstrual periods started in years, with extreme values pooled in groups of 9 years or less and 17 years or older. Age at natural menopause was defined as the age at which cessation of regular menstrual periods due to the body’s natural aging process occurred. In ARIC, women were asked, “Was your menopause natural or the result of surgery or radiation?” Only women who indicated natural menopause were included. Women in WHI who underwent hysterectomy, oophorectomy, or hormone replacement therapy before the onset of natural menopause were excluded. In both studies, women reporting age at natural menopause <40 years were excluded; women reporting age at natural menopause >60 years were censored at age 60. All women included in the present study were post-menopausal.

### Genotyping

Genotyping was performed on the Metabochip, a custom Illumina iSelect genotyping chip designed to genotype SNPs associated with metabolic traits and cardiovascular disease [43], [44]. The array also includes 2,207 SNPs associated at genome-wide significance to any trait published in the NHGRI GWAS catalog as of August 1, 2009. For each of these GWAS-identified SNPs, an additional proxy SNP with r^2^>0.90 in the CEU HapMap II dataset, plus up to four additional SNPs with r^2^>0.5 in the YRI HapMapII dataset were also included on the array. Lastly, SNPs selected to fine-map regions of interest related to metabolic traits, copy number variant-tagging SNPs, Major Histocompatibility Complex (MHC) SNPs, SNPs on the X and Y chromosomes, mitochondrial DNA SNPs, and “wildcard” SNPs were also targeted, for a total of approximately 200,000 SNPs. Of these, 161,098 (81.9%) passed quality control filters for tests of Hardy-Weinberg Equilibrium (>1×10^−7^) and genotyping efficiency (>95% call rate). There was no filter for minor allele frequency. The design and performance of this genotyping chip in this African American sample has been described in detail elsewhere [43].

### Statistical Analysis

All participants self-reported African American ancestry. To adjust for potential population stratification, we used the principal components method implemented in Eigenstrat [47]. We excluded any ancestry outliers further than eight standard deviations away from the mean for the first ten principal components determined by EIGENSOFT.

Linear regression was performed assuming an additive genetic model to test for associations between individual SNPs and the outcomes of age at menarche in years. We examined two models for menarche: 1) a minimally adjusted model that accounted only for study sites and principal components, and 2) a fully adjusted model that included study site, year of birth, principal components, and body mass index at ascertainment, with the understanding that BMI at ascertainment may be a poor proxy for BMI at age of menarche. Age at menarche was self-reported many years later at time of examination, which has been shown to be fairly accurate [48]. We studied one model for natural menopause using Cox’s proportional hazards for time-to-event (natural menopause) analysis, which adjusted for study site, principal components, and year of birth. Women with a missing age at menopause, an age at menopause <40 years, or hysterectomy, oophorectomy, or hormone replacement therapy after age 40 but prior to menopause, were excluded from the study. Women who had menopause >60 years had their ANM set as censored at age 60. A fixed effects meta-analysis was then performed using METAL to obtain effect size and standard error (SE) estimates [49]. All analyses were carried out in either METAL or the R software package, and data were plotted using LocusZoom [50], [51]. Statistical power to detect an expected association was estimated in Quanto [52] assuming the observed sample size and coded allele frequency in this African American cohort and the genetic effect size previously reported in the literature.

The overall goal of the study was to test for SNPs associated with AM and/or ANM using the Metabochip in African Americans from the WHI and ARIC studies. We looked to generalize to our population of African American women genes, gene regions (400 kb upstream and downstream of a gene of interest), and SNPs described in previous GWAS and candidate gene studies associated with AM and ANM. We tested all SNPs in the regions regardless of linkage disequilibrium (LD) with the index SNP, although we only considered a test of association generalized if the tested SNPs were identical to the index SNP or in strong LD with the index variant in HapMap CEU samples. For each candidate gene, we plotted results of single SNP tests of association using LocusZoom and examined regions 400 kb upstream and downstream of the gene/gene region of interest. Tests of association were considered significant for generalization at a liberal threshold of p<0.05. For previously reported variants not genotyped in our study, we identified SNPs in LD with our directly genotyped SNPs [53] and reported results from our minimally adjusted model (Model 1) for the proxy SNPs.

In addition to generalization, we sought to discover novel SNP-trait associations using the entire Metabochip. Significance in this discovery phase was defined as p<3.1×10^−07^, after Bonferroni correction (0.05/161,098). Because this threshold is highly conservative given the correlation among the SNPs on the Metabochip, we also defined an arbitrary suggestive significance level as p<1×10^−4^ in the discovery phase.

## Results

### Study Population

A total of 4,159 and 1,860 African American female participants met the study definitions for AM and ANM, respectively, and both PAGE studies were represented roughly equally (Table 1). In ARIC, the mean age at menarche was 12.9 years, which was slightly greater than the mean age at menarche in WHI (12.6 years) (Table 1). For ANM, the WHI group had a slightly later average onset than ARIC (Table 1).

**Table 1:** Study population characteristics of African American women from the PAGE Study.

		Age at Menarche (AM)		Age at Natural Menopause (ANM)	
		Study Population		Study Population	
		ARIC	WHI	ARIC	WHI
**Participants (n)**		2078	2081	994	866
**Age at menarche, yrs/Age at** **menopause, yrs**		12.89 (1.76)	12.56 (1.64)	47.97 (3.83)	50.84 (4.50)
**Age at enrollment, yrs**		53.36 (5.73)	61.01 (6.87)	53.07 (5.75)	61.30 (6.78)
**Body mass index, kg/m^2^**		30.86 (6.63)	31.34 (6.83)	31.29 (6.94)	30.95 (6.76)
**Weight, lbs.**		181.05 (39.68)	182.87 (41.26)	183.78 (40.80)	181.05 (40.63)
**Height, in.**		64.24 (2.43)	64.00 (2.63)	64.31 (2.38)	64.05 (2.75)
**Decade of birth, #(%)**	**1910s**	–	26 (1.24)	–	12 (1.39)
	**1920s**	504 (24.07)	414 (19.82)	221 (22.23)	183 (21.13)
	**1930s**	1083 (51.72)	981 (46.96)	522 (52.52)	414 (47.81)
	**1940s**	507 (24.21)	668 (31.98)	251 (25.25)	257 (29.68)

Data presented as means (sd) unless otherwise noted. Abbreviations: Atherosclerosis Risk in Communities (ARIC) and Women’s Health Initiative (WHI).

### Age at Menarche: Generalization to PAGE African Americans

To generalize previously-associated genetic variants in our African American population, we examined regions/genes previously associated with AM from either published candidate gene studies or GWAS: *CYP19A1, CYP17, CYP1B1*, *FTO, LIN28B*, 9q31.2 region, *IGF1, TNFSF11, TNFRSF11A,* and *LHCGR* [35]–[37], [54]–[59]. We also evaluated forty-two SNPs associated with AM identified in a recent meta-analysis by Elks *et al.* of >87,000 European-descent women from forty-nine studies [35].

**Figure 1:**
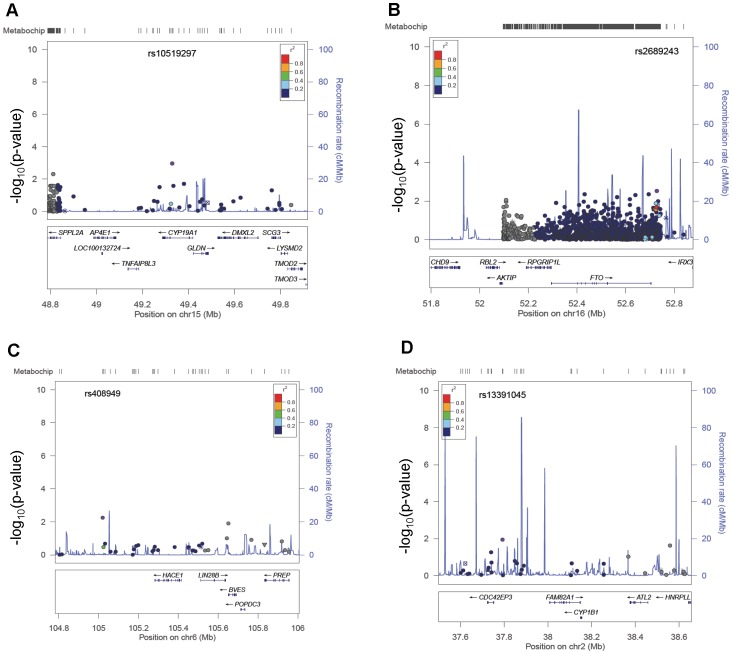
Regional Association Plots for Age at Menarche in African American women in the PAGE Study. Locus Zoom plots for selected gene regions in age at menarche analysis. Vertical axis is –log_10_ of the p-value, the horizontal axis is the chromosomal position. Each dot represents a SNP tested for association with age at natural menopause in 1,860 African American women from the PAGE Study. Approximate linkage disequilibrium between the most significant SNP, listed at the top of each plot, and the other SNPs in the plot is shown by the r^2^ legend in each plot. (A) Locus Zoom plot for the *CYP19A1* region, with rs10519297 the most significant SNP in the region. (B) Locus Zoom plot for the *FTO* region; rs2689243 was the most significant SNP in the plot region. (C) *LIN28B* region Locus Zoom plot; rs408949 was the most significant SNP in the plot region. (D) Locus Zoom plot of the *CYP1B1* region; rs13391045 was the most significant SNP in the plot region.

Overall, 11/21 (52%) SNPs previously identified for AM from earlier studies and 15/42 (36%) from the Elks *et al.* meta-analysis were directly genotyped or in strong (r^2^>0.70) LD in the CEU panel of HapMap with those genotyped (Table 2 and Table S1, respectively), and one generalized to this African American cohort: rs9385399, in LD with previously reported rs1361108 (r^2^ = 1.00, p = 0.01) (Table S1). Representative results of tests of association and LD in this African American sample are given for *CYP19A1, FTO, LIN28B,* and *CYP1B1*– genes previously associated with AM (Figure 1) [35], [36], [54], [60]. Three SNPs in *LIN28B* were included on the Metabochip (rs314277, rs4946651, and rs7759938), and while the direction of genetic effect was consistent with previous reports, all failed to reach statistical significance in this sample (p>0.30). Four additional SNPs in LD with these *LIN28B* SNPs were also not significant. At the 9q31 locus, rs7861820 and rs4452860, both located downstream of *TMEM38B,* had betas opposite to prior reports [36], [37]. Neither SNP nor their proxy SNPs were significant at p<0.05. Similarly, SNPs in LD (rs1856142 and rs605765) with previously associated variants in and around *FSHB* were not significantly associated with AM in this African American sample, though rs605765 (β = −0.06) had the same direction of effect and comparable magnitude as rs1782507 (β = −0.07) [59]. Results obtained under our fully adjusted model (Model 2) were similar to those of Model 1 and are available in Table 2.

**Table 2:** Comparison of GWAS-identified AM variants in African American women from the PAGE Study.

Locus		Gene/Region	Prior GWAS in European descent women				African American women from the PAGE Study						
SNP	Chr		Coded Allele	Beta	P-value	Ref.	Best Proxy SNP from present study	r^2^ in HapMap CEU/YRI	Coded Allele	Model 1		Model 2	
										Beta (SE)	P-value	Beta (SE)	P-value
rs314277	6	*LIN28B*	A	0.16	2.7E-13	[36]	rs314277	–	A	0.03(0.04)	0.34	0.03(0.04)	0.36
rs369065	6	*LIN28B*	C	0.11	2.4E-11	[36]	rs7759938	1.00/0.34	A	−0.02(0.04)	0.61	−0.02(0.04)	0.55
rs7759938	6	*LIN28B*	C	0.09	7.0E-09	[37]	rs7759938	–	A	−0.02(0.04)	0.61	−0.02(0.04)	0.55
rs314276	6	*LIN28B*	C	−0.22	1.5E-08	[57]	rs314274	1.00/0.73	A	0.05(0.04)	0.22	0.05(0.04)	0.24
rs314280	6	*LIN28B*	T	0.09	2.3E-08	[36], [39]	rs7759938	0.64/0.28	A	−0.02(0.04)	0.61	−0.02(0.04)	0.55
rs4946651	6	*LIN28B*	A	0.09	3.1E-08	[36]	rs4946651	–	A	0.03(0.04)	0.55	0.03(0.04)	0.55
rs314262	6	*LIN28B*	C	0.08	9.7E-08	[36]	rs7759938	0.60/0.29	A	−0.02(0.04)	0.61	−0.02(0.04)	0.55
rs7861820	9	9q31	C	−0.09	3.4E-09	[36]	rs7861820	–	A	−0.10(0.06)	0.10	−0.09(0.06)	0.12
rs12684013	9	9q31	T	−0.10	3.6E-08	[36]	rs4452860	0.81/0.01	A	−0.03(0.04)	0.43	−0.03(0.04)	0.42
rs4452860	9	9q31	G	−0.09	7.9E-08	[36]	rs4452860	–	A	−0.03(0.04)	0.43	−0.03(0.04)	0.42
rs7028916	9	9q31	A	−0.09	9.7E-08	[36]	rs4452860	0.98/0.85	A	−0.03(0.04)	0.43	−0.03(0.04)	0.42
rs2090409	9	9q31	A	−0.10	1.7E-09	[37]	rs4452860	0.83/0.82	A	−0.03(0.04)	0.43	−0.03(0.04)	0.42
rs555621	11	*FSHB*	C	0.06	0.001	[59]	rs1856142	0.43/0.71	A	0.03(0.04)	0.44	0.03(0.04)	0.36
rs1782507	11	*FSHB*	T	−0.07	0.006	[59]	rs605765	0.83/0.87	A	−0.06(0.04)	0.14	−0.06(0.04)	0.13
rs4953616	2	*LHCGR*	T	−0.07	0.006	[59]	rs1589749	0.17/0.05	A	0.002(0.07)	0.97	−0.01(0.07)	0.87
rs7579411	2	*LHCGR*	T	0.06	0.01	[59]	rs1589749	0.17/0.05	A	0.002(0.07)	0.97	−0.01(0.07)	0.87
rs4374421	2	*LHCGR*	C	0.06	0.02	[59]	rs17326321	0.19/0.69	A	−0.01(0.06)	0.86	−0.01(0.06)	0.84
rs2470144	15	*CYP19A1*	G	–	5.9E-06	[54]	rs12148492	0.23/0.01	A	−0.01(0.07)	0.91	−0.02(0.07)	0.73
rs2445761	15	*CYP19A1*	G	–	1.2E-06	[54]	rs4774585	0.28/0.02	A	0.04(0.05)	0.47	0.03(0.05)	0.58
rs9525641	13	*TNFSF11/RANKL*	T	–	0.04	[58]	rs931273	0.05/0.03	A	0.11(0.09)	0.24	0.11(0.09)	0.21
rs3826620	18	*TNFRSF11A/RANK*	A	–	0.02	[58]	rs8092336	0.16/0.22	A	0.16(0.17)	0.33	0.17(0.17)	0.29
rs6214	12	*IGF1*	G	–	0.02	[56]	rs6214	–	A	−0.01(0.04)	0.71	−0.02(0.04)	0.61

Comparison of previously reported SNPs associated with AM in European descent women to 4,159 African American women from the PAGE Study in a minimally adjusted model for AM (Model 1) and a model adjusted for study site, year of birth, principal components, and body mass index (Model 2). Data presented are for the previously identified SNP. If the previously identified SNP was not directly genotyped in present study, data shown are for the best proxy SNP based on linkage disequilibrium from the International HapMap Project CEU panel.

We also examined SNPs associated with AM that were reported in a recent meta-analysis performed by Elks *et al*. for the ReproGen Consortium [35]. Of the forty-two SNPs associated with AM in Elks *et al*., we detected an association with rs9385399 (p = 0.01), located downstream of *CENPW,* which is a perfect proxy (r^2^ = 1.00) for previously associated variant rs1361108, and the only SNP to generalize to our African American sample. We also identified an association with rs2947411 (p = 0.02) with AM (Table S1), though the directions of effect were opposite. One additional SNP, rs4929923 (p = 0.06), nearly reached the significance threshold and had a similar magnitude and direction of effect compared with the previous report. Overall, AM SNPs from previously published studies of European-descent women, including the Elks et al. meta-analysis, did not generalize to our PAGE African American population.

### Age at Natural Menopause: Generalization to PAGE African Americans

**Figure 2:**
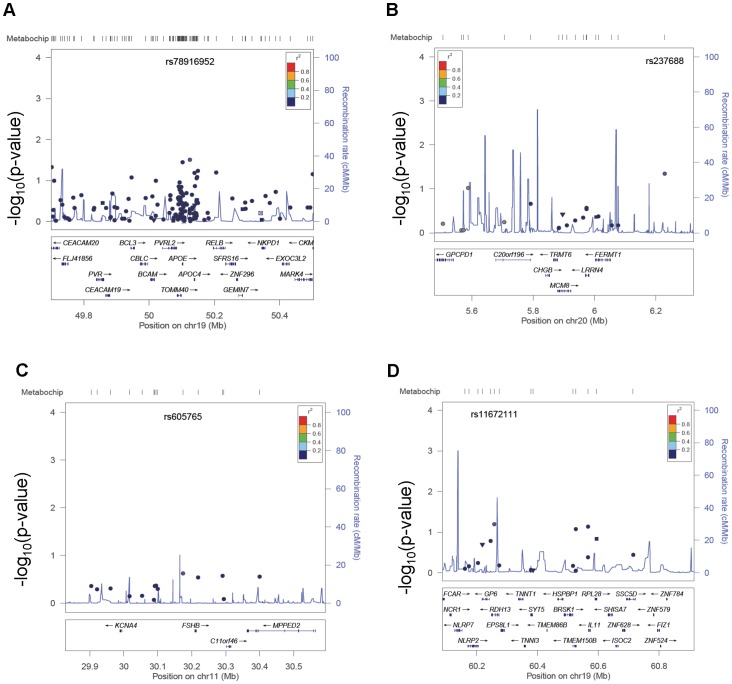
Regional Association Plots for Age at Natural Menopause in African American women in the PAGE Study. Locus Zoom plots for selected gene regions in age at natural menopause analysis. Vertical axis is the –log_10_ of the p-value, the horizontal axis is the chromosomal position. Each dot represents a SNP tested for association with age at natural menopause in 1,860 African American women from the PAGE Study. Linkage disequilibrium between the most significant SNP, listed at the top of each plot, and the other SNPs in the plot is shown by the r^2^ legend in each plot. (A) Locus Zoom plot for the *APOE* region, with rs78916952 the most significant SNP in the region. (B) Locus Zoom plot for the *MCM8* region; rs237688 is the most significant SNP in the plot region. (C) *FSHB* region Locus Zoom plot; rs605765 is the most significant SNP in the plot region. (D) Locus Zoom plot of the *BRSK1* region with rs11672111 as the most significant SNP in the plot region.

As with AM, to generalize results to our African American population, we examined previously identified 400 kb regions around genes associated with ANM from published candidate gene studies and GWAS: *APOE, CYP1B1, CYP19A1, CYP17A1*, *ESR2, BRSK1, FSHB, IGF2R, PPARG, TNFSF11, TNFRSF11A, BMP15, AMHR2, TMEM224, MCM8*, and *IGF1* (Figure 2, Table 3) [36], [38], [58], [59], [61]–[65]. We also examined twenty SNPs associated with ANM that were identified in a recent study by Stolk *et al.* [66] (Table S2).

**Table 3:** Comparison of GWAS-identified ANM variants in African American women in PAGE Study.

Locus		Gene/region	Prior GWAS in European descent women				African American women from the PAGE Study				
SNP	Chr		CodedAllele	Beta	P-value	Ref.	Best Proxy SNPfrom presentstudy	r^2^ in HapMapCEU/YRI	CodedAllele	Beta (SE)	P-value
rs16991615	20	*MCM8*	A	1.07	1.21E-21	[36], [62]	rs16991615	–	A	−0.17(0.15)	0.25
rs236114	20	*MCM8*	A	0.50	9.71E-11	[38]	rs236114	–	A	0.02(0.06)	0.69
rs1172822	19	*BRSK1*	T	−0.49	1.8E-19	[36], [38]	rs4806660	0.98/0.64	A	0.002(0.03)	0.97
rs2384687	19	*BRSK1*	C	−0.47	2.4E-18	[36]	rs11668309	0.85/0.43	A	0.02(0.04)	0.59
rs897798	19	*BRSK1*	G	−0.40	1.1E-14	[36]	rs8113016	0.72/0.02	A	0.12(0.05)	0.03
rs1065778	15	*CYP19A*	A	–	0.05	[61]	rs10519297	0.90/0.32	A	−0.01(0.05)	0.84
rs2255192	15	*CYP19A*	A	–	0.04	[61]	rs10459592	0.32/0.02	A	−0.02(0.04)	0.52
rs621686	11	*FSHB*	A	0.32	0.007	[59]	rs1856142	0.27/0.32	A	0.04(0.03)	0.29
rs7951733	11	*FSHB*	A	−0.32	0.02	[59]	rs7951733	–	A	0.11(0.13)	0.37
rs769450	19	*APOE*	A	–	0.007	[97]	rs769450	–	A	−0.07(0.03)	0.03
rs7412	19	*APOE*	–	–	0.001	[98]	rs7412	–	A	−0.03(0.05)	0.55
rs1019731	12	*IGF1*	C	−0.28	0.005	[59]	rs1019731	–	A	−0.03(0.11)	0.82
rs9457827	17	*IGF2R*	T	0.37	0.04	[59]	rs9457827	–	A	0.04(0.04)	0.28
rs4135280	3	*PPARG*	T	0.54	0.005	[59]	rs4135280	–	A	−0.14(0.18)	0.42
rs1256044	14	*ESR2*	G	–	0.03	[61]	rs1268656	0.08/0.004	A	−0.01(0.06)	0.88
rs1256059	14	*ESR2*	A	–	0.05	[61]	rs1268656	0.08/0.004	A	−0.01(0.06)	0.88
rs1056836	2	*CYP1B1*	G	–	0.04	[64]	rs10495874	0.04/0.03	A	−0.03(0.05)	0.60
rs346578	13	*TNFSF11*	A	–	0.007	[58]	rs6561072	0.07/0.07	A	0.04(0.04)	0.22
rs9525641	13	*TNFSF11*	T	–	0.01	[58]	rs931273	0.05/0.03	A	−0.02(0.08)	0.81
rs8086340	18	*TNFRSF11A*	G	–	0.02	[58]	rs8094440	0.10/0.01	A	0.03(0.03)	0.38
rs2002555	12	*AMHR2*	G	0.30	0.02	[65]	rs7131938	0.59/0.54	A	0.01(0.04)	0.84
rs2384687	19	*TMEM224*	C	0.38	1.39E-10	[38]	rs11668309	0.85/0.43	A	0.02(0.04)	0.59
rs897798	19	*TMEM224*	G	0.31	3.91E-08	[38]	rs8113016	0.72/0.02	A	0.12(0.05)	0.03

Comparison of previously reported SNPs associated with ANM in European and Chinese descent women to 1,860 African American women from the PAGE Study. Data presented are for the previously identified SNP. If the previously identified SNP was not directly genotyped in present study, data are shown for the best proxy SNP based on linkage disequilibrium calculated from the International HapMap Project CEU data.

Overall, 14/23 (40%) SNPs previously identified for ANM via GWAS and 6/20 SNPs from the Stolk *et al.* meta-analysis were directly genotyped on the Metabochip or were in strong LD (r^2^>0.70) in CEU panel of HapMap. 1/12 (8%) of the tested SNPs in these regions/genes generalized to this African American sample: rs8113016 (Table 3). Rs8113016, located in an intron of *TMEM150B/TMEM224* and downstream of *BRSK1*, is in LD with previously reported rs897798 (r^2^ = 0.72) and was associated with ANM in our sample (p = 0.03). An intronic *APOE* variant, rs769450, was associated with ANM (p = 0.03), though the nonsynonymous *APOE* rs7412 was not (p = 0.55); these SNPs are not in LD with each other (r^2^ = 0.04). In *BRSK1*, no previously reported SNPs were genotyped in our study; however, directly genotyped intronic *TMEM150B* rs4806660 was in very strong LD with intronic *BRSK1* rs1172822 (r^2^ = 0.98). *BRSK1* rs1168309, in strong LD with rs2384687 (r^2^ = 0.85) was not associated with ANM in this African American sample (p = 0.59).

Three of the twenty SNPs recently identified by Stolk *et al.* as associated with ANM were directly genotyped on the Metabochip. Two of the three genotyped SNPs (rs2303369 and rs2153157) had the same directions of effect, though the magnitudes were smaller. Of the remaining 17 SNPs not directly targeted by the Metabochip, three were in strong LD (HapMap CEU r^2^ ranging from 0.86 to 0.91) with the SNPs identified by Stolk *et al*: rs1176133, rs4668368, and rs12593363. For seven SNPs, no proxy SNP could be identified on the Metabochip (Table S2). Of the twenty SNPs identified in the Stolk *et al*. meta-analysis and directly or indirectly represented on the Metabochip, none were associated with ANM in this African American sample (Table S2).

### Age at Menarche: Discovery

We tested all SNPs genotyped on the Metabochip for an association with AM adjusted for study site and principal components (Model 1) and adjusted for study site, year of birth, principal components, and body mass index (Model 2) (Table 4). After accounting for multiple testing (p<3.1×10^−07^), no SNPs were significantly associated with AM in either model (Table S3). The most significant SNP in both models was rs11604207 (Model 1: p = 1.59×10^−06^; Model 2: p = 1.82×10^−06^), which is located upstream of *RSF1*, a gene encoding a chromatin remodeling protein implicated in ovarian and breast cancers [67]–[69] (Table S3).

**Table 4:** ANM Discovery–SNPs associated with age at natural menopause (ANM) in African American women from the PAGE Study.

CHR	SNP	GENE	GENE REGION	CODED ALLELE				
					CAF	BETA	SE	P VALUE
19	rs189596789	*LDLR*	upstream	A	0.006	1.09	0.20	4.98E-08
11	rs79972789	*KCNQ1*	intronic	C	0.997	−1.76	0.34	1.90E-07
5	rs181686584	*COL4A3BP*	intronic	A	0.002	2.35	0.46	2.85E-07
6	rs114158228	*CDKAL1*	intronic	A	9E-04	3.60	0.73	7.12E-07
21	rs117876865	*KCNE1*	downstream	A	9E-04	3.58	0.73	8.55E-07
10	rs11195485	*ADRA2A*	downstream	A	0.002	2.89	0.59	9.63E-07
11	rs11224401	*ARHGAP42*	intronic	A	0.997	2.20	0.45	1.13E-06
1	rs78937547	*SEC16B*	downstream	A	0.992	−1.97	0.41	1.89E-06
17	rs75394140	*KCNJ2*	downstream	A	0.002	−0.93	0.21	6.48E-06
11	rs76988592	*KCNJ1*	downstream	A	0.702	−0.93	0.21	7.24E-06
3	rs114451007	*PPARG*	intronic	A	0.253	1.70	0.38	9.30E-06
12	rs10846771	*DHX37*	downstream	A	0.997	−0.16	0.04	9.43E-06
11	rs12804247	*CCDC81*	upstream	A	0.655	0.17	0.04	1.45E-05
1	rs76571116	*SEC16B*	downstream	A	3E-04	−1.54	0.36	1.57E-05
17	rs17634167	*TTLL6*	cds-synon.	A	6E-04	−0.34	0.08	1.62E-05
7	rs117382431	*FKBP6*	downstream	A	0.999	4.38	1.03	2.17E-05
6	rs76294174	*LOC100130357*	intronic	C	3E-04	4.38	1.03	2.17E-05
6	rs74918542	*SCGN*	intronic	A	0.999	−4.38	1.03	2.17E-05
1	rs76078015	*NOS1AP*	intronic	A	9E-04	4.38	1.03	2.17E-05
18	rs117454233	*MC4R*	downstream	A	0.999	−4.38	1.03	2.17E-05
3	rs73025249	*PPARG*	intronic	A	9E-04	4.38	1.03	2.17E-05
3	rs182857216	*ETV5*	intronic	A	0.999	−4.38	1.03	2.17E-05
3	rs73027210	*PPARG*	intronic	A	9E-04	4.38	1.03	2.17E-05
9	rs75220302	*CDKN2A*	downstream	A	0.999	−4.38	1.03	2.18E-05
9	rs74599268	*CDKN2B*	upstream	A	3E-04	4.38	1.03	2.18E-05
9	rs3731245	*CDKN2A*	intronic	A	3E-04	4.38	1.03	2.18E-05
9	rs76774391	*CDKN2B*	upstream	C	3E-04	4.38	1.03	2.18E-05
2	rs117258126	*IRS1*	downstream	A	3E-04	4.38	1.03	2.18E-05
9	rs3808846	*CDKN2B*	5′ flanking	A	3E-04	4.38	1.03	2.18E-05
9	rs77706751	*CDKN2B*	upstream	A	6E-04	4.38	1.03	2.18E-05
9	rs3808845	*CDKN2B*	5′ flanking	A	3E-04	4.38	1.03	2.18E-05
9	rs76810097	*CDKN2B*	upstream	A	3E-04	4.38	1.03	2.18E-05
9	rs36228836	*CDKN2A*	5′ flanking	A	3E-04	4.38	1.03	2.18E-05
9	rs75039118	*ADAMTS13*	intronic	A	0.999	−4.38	1.03	2.19E-05
18	rs75914913	*MC4R*	downstream	A	3E-04	4.38	1.03	2.19E-05
11	rs190060931	*BUD13*	downstream	A	0.999	−4.38	1.03	2.21E-05
2	rs186397905	*IRS1*	downstream	C	3E-04	4.38	1.03	2.21E-05
16	rs9934222	*JPH3*	cds-synon.	A	0.163	−0.19	0.04	2.28E-05
15	rs72751410	*MAP2K5*	intronic	A	0.998	−1.51	0.36	2.30E-05
15	rs72747452	*LOC100506686*	intronic	A	0.002	1.51	0.36	2.30E-05
11	rs180751580	*NUCB2*	missense	C	0.999	−4.36	1.03	2.30E-05
3	rs186437034	*SCN5A*	intronic	A	0.999	−2.46	0.58	2.45E-05
7	rs78912482	*JAZF1*	upstream	A	0.012	0.64	0.15	3.04E-05
1	rs116071515	*SEC16B*	intronic	A	0.002	1.88	0.45	3.06E-05
6	rs1997770	*OFCC1*	downstream	A	0.970	−0.41	0.10	3.55E-05
7	rs118135044	*DGKB*	upstream	A	4E-04	4.22	1.02	3.73E-05
11	rs74402657	*ARFGAP2*	intronic	C	4E-04	2.93	0.72	3.96E-05
1	rs117217277	*SEC16B*	downstream	A	0.999	−2.97	0.72	3.97E-05
1	rs116881786	*SEC16B*	downstream	A	0.999	−2.97	0.72	3.97E-05
1	rs76471454	*SEC16B*	downstream	A	6E-04	2.97	0.72	3.97E-05
1	rs79775735	*SEC16B*	downstream	A	6E-04	2.97	0.72	3.97E-05
1	rs79468804	*SEC16B*	downstream	A	6E-04	2.97	0.72	3.97E-05
1	rs74703854	*SEC16B*	downstream	A	0.999	−2.97	0.72	3.97E-05
1	rs116923068	*SEC16B*	downstream	C	0.999	−2.97	0.72	3.97E-05
1	rs117674205	*SEC16B*	downstream	C	0.999	−2.97	0.72	3.97E-05
1	rs117260315	*SEC16B*	downstream	A	6E-04	2.97	0.72	3.97E-05
1	rs76020919	*SEC16B*	downstream	A	6E-04	2.97	0.72	3.97E-05
11	rs2306034	*LRP4*	UTR-3′	A	4E-04	2.94	0.72	3.99E-05
2	rs189110944	*IRS1*	downstream	A	4E-04	4.17	1.02	4.72E-05
5	rs1976311	*KCNN2*	upstream	C	0.996	−1.02	0.25	4.98E-05
7	rs13245084	*LOC100507421*	intronic	A	4E-04	4.14	1.02	5.07E-05
6	rs115178932	*LRRC16A*	intronic	A	4E-04	4.14	1.02	5.07E-05
1	rs77353590	*SYF2*	downstream	A	0.009	0.74	0.18	5.42E-05
2	rs111826230	*APOB*	upstream	A	0.984	−0.58	0.14	5.47E-05
11	rs193030163	*DDB2*	upstream	C	0.999	−4.11	1.02	5.57E-05
11	rs114702513	*KCNQ1*	intronic	A	0.996	−1.23	0.31	5.60E-05
6	rs117124693	*PHACTR1*	intronic	A	0.999	−4.11	1.02	5.62E-05
6	rs181947983	*SLC17A3*	upstream	A	4E-04	4.11	1.02	5.62E-05
15	rs183951867	*CHRNB4*	upstream	A	9E-04	4.11	1.02	5.62E-05
9	rs191930498	*CDKN2B*	upstream	C	4E-04	4.10	1.02	5.83E-05
17	rs192656758	*CCT6B*	downstream	A	4E-04	4.10	1.02	5.86E-05
7	rs740259	*JAZF1*	5′ flanking	A	4E-04	4.09	1.02	5.97E-05
1	rs114389068	*GPR153*	cds-synon.	A	0.005	0.93	0.23	6.07E-05
11	rs185476610	*KCNQ1*	intronic	A	0.999	−4.08	1.02	6.24E-05
16	rs246192	*NDRG4*	intronic	C	0.256	0.15	0.04	6.25E-05
7	rs192457106	*JAZF1*	intronic	A	0.999	−4.08	1.02	6.35E-05
7	rs73702566	*WBSCR22*	intronic	A	0.999	−4.08	1.02	6.35E-05
6	rs187190790	*TAP2D*	upstream	A	0.999	−4.08	1.02	6.38E-05
7	rs74984879	*DGKB*	upstream	C	0.999	−2.04	0.51	6.40E-05
11	rs184056970	*ARAP1*	intronic	A	4E-04	4.07	1.02	6.53E-05
3	rs76909367	*COLQ*	intronic	A	4E-04	4.06	1.02	6.89E-05
10	rs11187795	*PLCE1*	intronic	A	4E-04	4.06	1.02	6.93E-05
6	rs186129489	*TFAP2D*	intronic	A	4E-04	4.05	1.02	7.12E-05
2	rs73923981	*BRE*	intronic	A	9E-04	4.05	1.02	7.32E-05
15	rs180807356	*ADAMTS7*	upstream	A	0.999	−4.04	1.02	7.52E-05
5	rs10062135	*NPR3*	intronic	A	0.009	0.73	0.19	7.85E-05
12	rs17568045	*C12orf42*	intronic	A	0.993	−0.86	0.22	8.11E-05
1	rs116411856	*WARS2*	upstream	A	0.003	1.32	0.34	8.16E-05
1	rs78696400	*LYPLAL1*	downstream	A	0.985	−0.58	0.15	8.96E-05
15	rs74979292	*C15orf39*	upstream	A	0.002	1.49	0.38	9.29E-05
11	rs144204188	*TRIM66*	intronic	A	0.002	2.79	0.72	9.39E-05
1	rs78411379	*TBX15*	intronic	A	0.999	−2.27	0.58	9.62E-05
15	rs190893945	*ADAMTSL3*	intronic	A	0.998	−1.76	0.45	9.67E-05
9	rs12555547	*CDKN2B*	upstream	C	0.998	−2.30	0.59	9.69E-05
2	rs10932320	*C2orf67*	intronic	A	0.807	−0.17	0.04	9.93E-05

Tests of association at p≤1E-04 from single SNP linear regressions adjusted for study site and principal components in 1,860 African American women from the PAGE Study are shown. For each significant test of association, the chromosome, rs number, nearest gene, location, coded allele, beta, standard error (SE), and p-value are given. Genes listed are nearest genes to the SNP as measured from the transcription start site for upstream SNPs or the transcription stop site for downstream SNPs. Abbreviations: CAF, coded allele frequency.

### Age at Natural Menopause: Discovery

We tested all SNPs on the Metabochip for associations with ANM adjusted for study site and principal components. Three SNPs were significant after Bonferroni correction (p<3.1×10^−07^): *LDLR* (rs189596789, p = 4.98×10^−08^), *KCNQ1* (rs79972789, p = 1.90×10^−07^), and *COL4A3BP* (rs181686584, p = 2.85×10^−07^). The most significant association was with rs189596789, located approximately 10 kb upstream of the low-density lipoprotein receptor (*LDLR*) gene, which has been associated with familial hypercholesterolemia [70], [71]. Several of the most significant SNPs for ANM were located in/near genes previously associated with obesity, type 2 diabetes (T2D), coronary artery disease and lipid metabolism, e.g., *LDLR* (rs189596789), *NOS1AP* (rs76078015), *DGKB* (rs74486449), *LYPLAL1* (rs78696400), and *CDKAL1* (rs114158228) (Table 4). We were unable to generalize the previously reported association between ANM and *PPARG* rs4135280 in this African American sample.

Two genes were suggestively associated with both ANM and AM at a nominal significance threshold. *PHACTR1* was suggestively associated with AM (rs73725617; Table S3) and ANM (rs117124693; Table 4). Though the direction of effects was similar for each SNP in *PHACTR1*, the SNPs are not in LD with each other. Likewise, SNPs in *ARHGAP42*, located at the 11q22.1 locus, were suggestively associated with AM (rs11224447; Table S3) and ANM (rs11224401; Table 4), but are not in LD with each other, though the direction of effects was the same.

## Discussion

Here we demonstrated the use of the Metabochip genotyping array to identify SNPs associated with AM and ANM in a sample of African American women. Previous GWAS studies for AM and ANM have been performed in primarily European descent populations; generalization to diverse populations has largely been lacking [72]. Our study is the first, to our knowledge, to consider this trait in a large African American cohort. We were able to generalize only one previously identified variant for AM and two variants for ANM to our African American cohort [AM: rs1361108; ANM: rs897798 and rs9385399 (proxy for rs1361108)]. Overall, however, we were unable to generalize the majority of significant associations for previously identified SNPs associated with AM, including *LIN28B* or the 9q31 locus, or with ANM, including *MCM8* or *TMEM150b/TMEM224*, which have recently been identified in several GWAS of European-descent women. Our inability to replicate earlier findings in our African American sample may have, in part, resulted from scant Metabochip coverage of these regions. The emphasis of the Metabochip on genes involved in lipid metabolism and cardiovascular traits is evident comparing coverage in the *FTO* region (1053 SNPs) to the *LIN28B* region (28 SNPs).

In the discovery phase of our AM analysis, none of our results reached genome-wide significance. However, the ANM analysis yielded three associations that were significant after multiple testing corrections. Broadly, we demonstrate the ability to potentially uncover new variants associated with age at natural menopause in our African American cohort using the Metabochip.

Several studies have shown relationships between a woman’s reproductive milestones (AM, ANM, parity) and menstrual characteristics and risk for breast cancer, endometrial cancer, and ovarian cancer [73]–[77] and chronic diseases such as diabetes, osteoporosis and cardiovascular disease (briefly [78]–[80] ). The most significant result in the ANM analysis was a SNP located upstream of *LDLR* (rs189596789) which encodes a low density lipoprotein receptor implicated in familial cholesterolemia. *KCNQ1* (rs79972789) also reached genome wide significance in our ANM analysis. Numerous variants in *KCNQ1* have also been implicated in type 2 diabetes in several populations, though none were in linkage disequilibrium with rs79972789 [81]–[86]. Recently, Buber *et al.* evaluated the role of menopausal hormonal changes with cardiac events in women with mutations in *KCNQ1* and congenital long-QT syndrome (LQTS) [87] and determined the onset of menopause was associated with an increase in the risk of cardiac events in LQTS women. Though not significant, suggestive AM associations included *LPL* and *CYP4F22*, which are associated with type 2 diabetes and lipid metabolism (rs1372339, rs4922116, rs1273516), and *TMEM18* (rs2947411), associated with obesity and body mass index [88], [89]. These ANM associations and suggestive AM associations with genes involved in cardiovascular function, lipid metabolism, and type 2 diabetes concur with research showing later AM lowers obesity and diabetes risk while earlier ANM increases risk for cardiovascular disease, obesity and insulin resistance [90], [91].

Different pathways appear to be involved in the initiation and cessation of menses. Prior GWAS and linkage studies performed in European descent or Asian populations for AM and ANM show little concordance with specific genes (reviewed in [5]). Our analysis is consistent with this observation. Only *PHACTR1* and *ARHGAP42* SNPs were suggestively significant in both our AM and ANM analyses. *PHACTR1* is a phosphatase and actin regulator which has been implicated in coronary artery disease [92], [93]. Its role in menarche and menopause is yet to be determined. *ARHGAP42*, a Rho GTPase activating protein, has not yet been evaluated for a role in menarche or menopause. A GWAS identified intronic *ARHGAP42* rs633185 is associated with blood pressure [94], but this variant is not in strong LD with *ARHGAP42* variants suggestively associated with either AM or ANM in this study. A recent study by Lu *et al.*, found SNPs in both *TNFSF11* and *TNFRSF11A* significant for AM and ANM [58]. SNPs genotyped on the Metabochip were in weak LD with the reported SNPs and failed to reach significance in this African American sample. Given the role that both *PHACTR1* and *ARHGAP42* play in atherosclerosis, osteoporosis and the development of lactation glands in pregnancy, further investigation on the influence of these genes in AM and ANM is warranted [95], [96].

The Metabochip was designed to be a cost-effective method of genotyping approximately 200,000 metabolic and cardiovascular SNPs and SNPs in other useful regions of the genome, such as the HLA region and the X and Y chromosomes. Overall, median SNP density on the Metabochip is approximately one SNP per 370 bases [43]. This coverage appears sufficient to replicate some loci associated with both cardiovascular or metabolic traits and AM/ANM. However, we found instances of previously identified genes for AM/ANM with little/no Metabochip coverage (*CYP1B1, LIN28B, ESR2, and BRSK1*) which may have impacted our results. Additionally, prior studies that identified SNPs associated with AM and ANM were performed primarily in European-descent cohorts. Though our study included over 4,000 African American women, we had limited power to identify significant associations in most previously identified loci, which may explain why we failed to detect the same associations identified in European-descent GWAS. For specific tests of association, our power was impacted by sample size and by minor allele frequencies. For example, the allele frequency for rs7861820 in this African American cohort was 0.11 compared to a higher frequency observed in HapMap CEU (0.57; Table S4). Interestingly, we were adequately powered (>98%) to generalize the intronic *LIN28B* SNP, rs314277, with AM in our sample, yet failed to find an association with this SNP or with SNPs in strong LD with it.

Metabochip performance in non-European populations was recently evaluated in a pilot study in African American PAGE participants [43]. In this pilot study, Buyske *et al*. demonstrated that the majority (89%) of SNPs targeted by the Metabochip passed rigorous quality control with high call rates [43]. Using lipid traits as an example, Buyske *et al*. demonstrated that Metabochip data can be used to replicate known GWAS-identified SNP-trait relationships. Furthermore, the pilot study demonstrated that Metabochip data can be used to fine-map GWAS-identified regions to uncover potential novel index SNPs specific to African Americans in an established locus for that trait. Fine-mapping data for AM/ANM was not included in the Metabochip content. While we were able to use the Metabochip to identify potentially novel SNP-trait relationships for AM/ANM, additional fine-mapping efforts of other loci already implicated for these traits are needed. Furthermore, additional studies in general are warranted for diverse (non-European descent) populations using Metabochip or other arrays designed for fine-mapping. Admixture in the African American population and its associated decreased LD compared to European Americans challenge identification of trait-associated SNPs. Targeted fine mapping, such as use of the Metabochip, may be more appropriate in some circumstances than GWAS to evaluate specific SNPs and regions associated with particular traits.

Although the Metabochip was designed for genotyping of cardiovascular and metabolic SNPs, this study demonstrates the feasibility of utilizing such a targeted chip to identify SNP associations with age at menarche and age at natural menopause. We identified potentially novel associations with AM/ANM at loci implicated in cardiovascular traits, obesity and cancer. This may result from pleiotropic loci or may suggest that the AM/ANM timing mechanisms influence underlying disease process. With numerous genes implicated in both metabolic and cardiovascular phenotypes and both AM and ANM, further studies will allow us to consider how specific genes may influence the reproductive lifespan in women.

## Supporting Information

Table S1 **Comparison of SNPS in Elks et al. meta-analysis for AM to African American women in PAGE Study.**
(DOCX)

Table S2 **Comparison of SNPs in Stolk et al. meta-analysis for ANM to African American women in PAGE Study.**
(DOCX)

Table S3 **AM Discovery—SNPs associated (p<1e−4) with AM in African American women from the PAGE Study.**
(DOCX)

Table S4 **Minor allele frequency comparisons of African American women in PAGE Study to HapMap CEU Panel.**
(DOCX)
