# Supplementary material for: Genetic Variation and Reproductive Timing: African American Women from the Population Architecture Using Genomics and Epidemiology (PAGE) Study
Source: PLoS One. 2013 Feb 12;8(2):e55258. doi: 10.1371/journal.pone.0055258 (PMC3570525; doi:10.1371/journal.pone.0055258)
Supplement: Table S1 — Comparison of SNPS in Elks et al. meta-analysis for AM to African American women in PAGE Study. (DOCX) [file pone.0055258.s001.docx]

**Table S1:** **Comparison of SNPs in Elks et al. meta-analysis for AM to African American women in PAGE Study.**

| **Locus** | | **Gene/**  **region** | **Elks et al.** | | | | **African American women from the PAGE Study** | | | | | |
| --- | --- | --- | --- | --- | --- | --- | --- | --- | --- | --- | --- | --- |
| **SNP** | **Chr** |  | **Minor Allele** | **MAF** | **Beta** | **P-value** | **Best Proxy SNP from present study** | **r^2^ in HapMap CEU/YRI** | **Coded Allele** | **CAF** | **Beta (SE)** | **P-value** |
| rs7759938 | 6 | *LIN28B* | C | 0.32 | 0.12 | 5.4E-60 | rs7759938 | - | A | 0.46 | -0.02(0.04) | 0.61 |
| rs2090409 | 9 | *TMEM38B* | A | 0.31 | -0.09 | 2.2E-33 | rs4452860 | 0.83/0.82 | A | 0.67 | -0.03(0.04) | 0.43 |
| rs1079866 | 7 | *INHBA* | G | 0.15 | 0.08 | 5.5E-14 | rs6947337 | 0.02/0.001 | A | 0.28 | -0.07(0.04) | 0.10 |
| rs466639 | 1 | *RXRG* | T | 0.13 | -0.08 | 1.3E-13 | rs285482 | 0.36/0.05 | A | 0.55 | -0.003(0.04) | 0.93 |
| rs6438424 | 3 | *3q13.3* | A | 0.50 | -0.05 | 1.4E-13 | rs9283566 | 0.51/0.15 | A | 0.29 | 0.03(0.04) | 0.47 |
| rs1398217 | 18 | *FUSSEL18* | G | 0.43 | -0.05 | 2.3E-13 | NA | NA | NA | NA | NA | NA |
| rs12617311 | 2 | *PLCL1* | A | 0.32 | -0.06 | 6.0E-13 | NA | NA | NA | NA | NA | NA |
| rs9635759 | 17 | *CA10* | A | 0.32 | 0.06 | 7.3E-13 | NA | NA | NA | NA | NA | NA |
| rs6589964 | 11 | *BSX* | A | 0.48 | -0.05 | 1.9E-12 | rs922252 | 0.24/0.16 | A | 0.40 | 0.03(0.04) | 0.43 |
| rs10980926 | 9 | *ZNF483* | A | 0.36 | 0.05 | 4.2E-11 | rs6477828 | 0.12/0.13 | A | 0.55 | -0.01(0.04) | 0.76 |
| rs17268785 | 2 | *CCDC85A* | G | 0.17 | 0.06 | 9.7E-11 | NA | NA | NA | NA | NA | NA |
| rs13187289 | 5 | *PHF15* | G | 0.20 | 0.06 | 1.9E-10 | NA | NA | NA | NA | NA | NA |
| rs7642134 | 3 | *VGLL3* | A | 0.38 | -0.05 | 3.5E-10 | rs1825896 | 0.02/0.05 | A | 0.15 | -0.04(0.05) | 0.40 |
| rs17188434 | 2 | *NR4A2* | C | 0.07 | -0.09 | 1.1E-09 | NA | NA | NA | NA | NA | NA |
| rs2002675 | 3 | *TRA2B* | G | 0.42 | 0.04 | 1.2E-09 | NA | NA | NA | NA | NA | NA |
| rs7821178 | 8 | *PXMP3* | A | 0.34 | -0.05 | 3.0E-09 | NA | NA | NA | NA | NA | NA |
| rs1659127 | 16 | *MKL2* | A | 0.34 | 0.05 | 4.0E-09 | rs1659127 | - | A | 0.30 | 0.03(0.04) | 0.46 |
| rs10423674 | 19 | *CRTC1* | A | 0.35 | 0.04 | 5.9E-09 | rs757318 | 0.63/0.14 | A | 0.78 | -0.03(0.04) | 0.49 |
| rs10899489 | 11 | *GAB2* | A | 0.15 | 0.06 | 8.1E-09 | rs7115850 | 0.96/0.58 | C | 0.58 | -0.05(0.04) | 0.23 |
| rs6575793 | 14 | *BEGAIN* | C | 0.42 | 0.04 | 1.2E-08 | NA | NA | NA | NA | NA | NA |
| rs4929923 | 11 | *TRIM66* | T | 0.36 | 0.04 | 1.2E-08 | rs4929923 | - | A | 0.46 | 0.07(0.04) | 0.06 |
| rs6439371 | 3 | *TMEM108* | G | 0.34 | 0.04 | 1.3E-08 | NA | NA | NA | NA | NA | NA |
| rs900145 | 11 | *ARNTL* | C | 0.30 | 0.04 | 1.6E-08 | rs900145 | - | A | 0.47 | -0.03(0.04) | 0.41 |
| rs6762477 | 3 | *RBM6* | G | 0.44 | 0.05 | 1.6E-08 | rs2240327 | 0.69/0.16 | A | 0.64 | -0.06(0.04) | 0.15 |
| rs2947411 | 2 | *TMEM18* | A | 0.17 | 0.05 | 1.7E-08 | rs2947411 | - | A | 0.23 | -0.10(0.04) | 0.02 |
| rs1361108 | 6 | *C6orf173* | T | 0.46 | -0.04 | 1.7E-08 | rs9385399 | 1.00/0.60 | A | 0.25 | -0.12(0.04) | 0.01 |
| rs1364063 | 16 | *NFAT5* | C | 0.43 | 0.04 | 1.8E-08 | rs889398 | 0.93/0.42 | A | 0.28 | -0.05(0.04) | 0.25 |
| rs633715 | 1 | *SEC16B* | C | 0.20 | -0.05 | 2.1E-08 | rs516636 | 1.00/0.92 | A | 0.11 | 0.04(0.06) | 0.55 |
| rs4840086 | 6 | *PRDM13* | G | 0.42 | -0.04 | 2.4E-08 | NA | NA | NA | NA | NA | NA |
| rs7617480 | 3 | *KLHDC8B* | A | 0.22 | 0.05 | 2.8E-08 | rs13096474 | 0.76/0.56 | A | 0.35 | -0.05(0.04) | 0.21 |
| rs9939609 | 16 | *FTO* | A | 0.40 | -0.04 | 3.1E-08 | rs9939609 | - | A | 0.48 | 0.003(0.04) | 0.93 |
| rs852069 | 20 | *PCSK2* | A | 0.37 | -0.04 | 3.3E-08 | NA | NA | NA | NA | NA | NA |
| rs757647 | 5 | *KDM3B* | A | 0.22 | -0.05 | 5.4E-08 | rs757647 | - | A | 0.41 | -0.06(0.04) | 0.09 |
| rs9555810 | 13 | *C13orf16* | G | 0.28 | 0.04 | 5.6E-08 | NA | NA | NA | NA | NA | NA |
| rs16938437 | 11 | *PHF21A* | T | 0.09 | -0.07 | 5.9E-08 | rs16938437 | - | A | 0.23 | 0.02(0.04) | 0.63 |
| rs2687729 | 3 | *EEFSEC* | G | 0.27 | 0.04 | 1.3E-07 | rs2811415 | 0.17/0.83 | A | 0.29 | 0.05(0.04) | 0.26 |
| rs1862471 | 19 | *OLFM2* | G | 0.47 | 0.04 | 1.5E-07 | NA | NA | NA | NA | NA | NA |
| rs12472911 | 2 | *LRP1B* | C | 0.20 | 0.05 | 1.5E-07 | NA | NA | NA | NA | NA | NA |
| rs3914188 | 3 | *ECE2* | G | 0.27 | -0.04 | 2.6E-07 | NA | NA | NA | NA | NA | NA |
| rs2243803 | 18 | *SLC14A2* | A | 0.40 | 0.04 | 3.4E-07 | NA | NA | NA | NA | NA | NA |
| rs3743266 | 15 | *RORA* | C | 0.32 | -0.04 | 8.0E-07 | rs17270188 | 0.31/0.02 | A | 0.92 | 0.05(0.07) | 0.47 |
| rs7359257 | 15 | *IQCH* | A | 0.45 | 0.03 | 1.9E-06 | rs7359257 | - | A | 0.66 | 0.06(0.04) | 0.15 |

Comparison of previously reported SNPs associated with AM in European descent women to 4,159 African American women from the PAGE Study in a minimally adjusted for study site and principal components for AM (Model 1). Beta values from Elks *et al*. converted from weeks to years [1]. Data presented are for the previously identified SNP. If the previously identified SNP was not directly genotyped in present study, data shown are for the best proxy SNP based on linkage disequilibrium from the International HapMap Project CEU panel. (NA)= no sufficient proxy available on the Metabochip. Abbreviations: CAF Coded Allele Frequency.

Reference List

1. Elks CE, Perry JR, Sulem P, Chasman DI, Franceschini N, et al. (2010) Thirty new loci for age at menarche identified by a meta-analysis of genome-wide association studies. Nat Genet 42: 1077-1085. ng.714 [pii];10.1038/ng.714 [doi].
