# Supplementary material for: Genetic Variation and Reproductive Timing: African American Women from the Population Architecture Using Genomics and Epidemiology (PAGE) Study
Source: PLoS One. 2013 Feb 12;8(2):e55258. doi: 10.1371/journal.pone.0055258 (PMC3570525; doi:10.1371/journal.pone.0055258)
Supplement: Table S2 — Comparison of SNPs in Stolk et al. meta-analysis for ANM to African American women in PAGE Study. (DOCX) [file pone.0055258.s002.docx]

**Table S2: Comparison of SNPs in Stolk *et al*. meta-analysis for ANM to African American women in PAGE Study.**

| **Locus** | | **Gene/**  **region** | **Stolk et al.** | | | | **African American women from the PAGE Study** | | | | | |
| --- | --- | --- | --- | --- | --- | --- | --- | --- | --- | --- | --- | --- |
| **SNP** | **Chr** |  | **Minor Allele** | **MAF** | **Beta** | **P-value** | **Best Proxy SNP from present study** | **r^2^ in HapMap CEU/YRI** | **Coded allele** | **CAF** | **Beta (SE)** | **P-value** |
| rs4246511 | 1 | *RHBDL2* | T | 0.27 | 0.24 | 9.08E-17 | NA | NA | NA | NA | NA | NA |
| rs1635501 | 1 | *EXO1* | C | 0.48 | -0.16 | 8.46E-10 | rs1776133 | 0.91/0.23 | A | 0.70 | -0.05(0.04) | 0.17 |
| rs2303369 | 2 | *FNDC4* | T | 0.39 | -0.18 | 2.25E-12 | rs2303369 | - | A | 0.36 | -0.02(0.03) | 0.48 |
| rs10183486 | 2 | *TLK1* | T | 0.37 | -0.20 | 2.21E-14 | rs4668368 | 0.86/0.62 | A | 0.65 | 0.03(0.03) | 0.37 |
| rs7606918 | 2 | *METAP1D* | G | 0.16 | -0.23 | 2.89E-08 | rs11681005 | 0.08/0.02 | A | 0.13 | -0.06(0.05) | 0.25 |
| rs4693089 | 4 | *HELQ* | G | 0.49 | 0.23 | 2.38E-19 | NA | NA | NA | NA | NA | NA |
| rs890835 | 5 | *RNF44* | A | 0.11 | 0.18 | 6.10E-06 | NA | NA | NA | NA | NA | NA |
| rs365132 | 5 | *UIMC1* | T | 0.49 | 0.29 | 9.11E-32 | NA | NA | NA | NA | NA | NA |
| rs2153157 | 6 | *SYCP2L* | A | 0.49 | 0.17 | 7.76E-12 | rs2153157 | - | A | 0.70 | 0.03(0.04) | 0.47 |
| rs1046089 | 6 | *PRRC2A* | A | 0.35 | -0.21 | 1.63E-16 | rs9264532 | 0.08/0.05 | A | 0.65 | 0.02(0.03) | 0.50 |
| rs2517388 | 8 | *ASH2L* | G | 0.17 | 0.26 | 9.31E-15 | rs4976896 | 0.003/0.000 | A | 0.78 | 0.01(0.04) | 0.77 |
| rs12294104 | 11 | *MPPED2* | T | 0.17 | 0.23 | 1.46E-11 | rs7951733 | 0.35/- | A | 0.99 | 0.11(0.13) | 0.37 |
| rs2277339 | 12 | *PRIM1* | G | 0.10 | -0.38 | 2.47E-19 | rs12809466 | 0.01/0.04 | A | 0.88 | 0.02(0.05) | 0.66 |
| rs3736830 | 13 | *KPNA3* | G | 0.16 | -0.18 | 9.41E-08 | NA | NA | NA | NA | NA | NA |
| rs4886238 | 13 | *TDRD3* | A | 0.33 | 0.17 | 9.53E-11 | NA | NA | NA | NA | NA | NA |
| rs2307449 | 15 | *POLG* | G | 0.41 | -0.18 | 3.56E-13 | rs12593363 | 0.91/0.12 | A | 0.74 | 0.01(0.04) | 0.83 |
| rs10852344 | 16 | *GSPT1* | C | 0.42 | 0.17 | 1.01E-11 | rs8053435 | 0.04/0.01 | A | 0.77 | -0.01(0.04) | 0.77 |
| rs11668344 | 19 | *TMEM150B* | G | 0.36 | -0.42 | 1.45E-59 | NA | NA | NA | NA | NA | NA |
| rs12461110 | 19 | *NLRP11* | A | 0.36 | -0.16 | 8.74E-10 | rs302469 | 0.03/0.004 | A | 0.25 | -0.05(0.04) | 0.22 |
| rs16991615 | 20 | *MCM8* | A | 0.07 | 0.95 | 1.42E-73 | rs16991615 | - | A | 0.01 | -0.17(0.15) | 0.25 |

Comparison of previously reported SNPs from Stolk *et al.* meta-analysis [2] associated with ANM in a combined cohort (discovery and replication) of 53,403 European descent women to 1,860 PAGE Study African American women in a minimally adjusted for study site and principal components for ANM. MAF from Stolk *et al*. reported for discovery cohort, beta and p-values reported for combined discovery and replication cohorts [2]. Data presented are for the previously identified SNP. If the previously identified SNP was not directly genotyped in present study, data shown are for the best proxy SNP based on linkage disequilibrium from the International HapMap Project CEU panel. (NA)= no sufficient proxy available on the Metabochip.

Reference List

1. Stolk L, Perry JR, Chasman DI, He C, Mangino M, Sulem P, et al. (2012) Meta-analyses identify 13 loci associated with age at menopause and highlight DNA repair and immune pathways. Nat Genet . ng.1051 [pii];10.1038/ng.1051 [doi].
