# Supplementary material for: Genetic Variation and Reproductive Timing: African American Women from the Population Architecture Using Genomics and Epidemiology (PAGE) Study
Source: PLoS One. 2013 Feb 12;8(2):e55258. doi: 10.1371/journal.pone.0055258 (PMC3570525; doi:10.1371/journal.pone.0055258)
Supplement: Table S3 — AM Discovery—SNPs associated (p<1e−4) with AM in African American women from the PAGE Study. (DOCX) [file pone.0055258.s003.docx]

**Table S3:** **AM Discovery—SNPs associated (p<1e-4) with age at menarche (AM) in African American women from the PAGE Study.**

| **CHR** | **SNP** | **GENE** | **GENE REGION** | **CODED ALLELE** |  | **MODEL 1** | | | **MODEL 2** | | |
| --- | --- | --- | --- | --- | --- | --- | --- | --- | --- | --- | --- |
|  |  |  |  |  | **CAF** | **BETA** | **SE** | **P VALUE** | **BETA** | **SE** | **P VALUE** |
| 11 | rs11604207 | *RSF1* | upstream | A | 0.58 | 0.38 | 0.08 | 1.59E-06 | 0.38 | 0.08 | 1.82E-06 |
| 17 | rs59622946 | *HEXIM2* | flanking | A | 0.01 | 1.01 | 0.23 | 1.14E-05 | 1.04 | 0.23 | 4.93E-06 |
| 1 | rs2753399 | *ZFYVE9* | intronic | A | 0.08 | 0.31 | 0.07 | 1.16E-05 | 0.30 | 0.07 | 1.63E-05 |
| 5 | rs40602 | *MAST4* | intronic | A | 0.57 | 0.16 | 0.04 | 1.26E-05 | 0.15 | 0.04 | 3.65E-05 |
| 15 | rs7181548 | *C15orf27* | intronic | A | 0.58 | -0.16 | 0.04 | 1.96E-05 | -0.16 | 0.04 | 2.36E-05 |
| 8 | rs4922116 | *LPL* | downstream | A | 0.15 | -0.22 | 0.05 | 2.18E-05 | -0.21 | 0.05 | 3.10E-05 |
| 8 | rs1372339 | *LPL* | downstream | A | 0.84 | 0.21 | 0.05 | 2.19E-05 | 0.21 | 0.05 | 2.89E-05 |
| 17 | rs116523982 | *HEXIM2* | flanking | A | 0.01 | 0.96 | 0.23 | 2.51E-05 | 0.99 | 0.23 | 1.11E-05 |
| 17 | rs3744412 | *HEXIM2* | 5’ UTR | C | 0.01 | 0.96 | 0.23 | 2.52E-05 | 1.00 | 0.23 | 1.12E-05 |
| 3 | rs11922097 | *PPP2R3A* | upstream | A | 0.55 | 0.16 | 0.04 | 2.55E-05 | 0.16 | 0.04 | 1.64E-05 |
| 17 | rs16939893 | *HEXIM2* | intronic | A | 0.004 | 1.26 | 0.30 | 2.73E-05 | 1.29 | 0.30 | 1.46E-05 |
| 6 | rs73725617 | *PHACTR1* | intronic | A | 0.99 | -0.64 | 0.15 | 3.11E-05 | -0.60 | 0.15 | 6.83E-05 |
| 7 | rs11979121 | *TFEC* | upstream | A | 0.98 | -0.52 | 0.13 | 4.50E-05 | -0.51 | 0.13 | 5.41E-05 |
| 3 | rs1320623 | *LSG1* | intronic | A | 0.37 | 0.15 | 0.04 | 5.64E-05 | 0.15 | 0.04 | 5.56E-05 |
| 12 | rs61507607 | *CUX2* | intronic | A | 0.43 | 0.15 | 0.04 | 5.85E-05 | 0.13 | 0.04 | 3.15E-04 |
| 11 | rs11224447 | *ARHGAP42* | intronic | A | 0.07 | 0.30 | 0.07 | 6.11E-05 | 0.30 | 0.07 | 6.11E-05 |
| 19 | rs1273516 | *CYP4F22* | downstream | A | 0.40 | 0.15 | 0.04 | 6.33E-05 | 0.15 | 0.04 | 6.29E-05 |
| 6 | rs9503555 | *IRF4* | upstream | A | 0.78 | 0.18 | 0.04 | 6.83E-05 | 0.18 | 0.04 | 6.03E-05 |
| 15 | rs8032832 | *FAM174B* | upstream | A | 0.35 | -0.15 | 0.04 | 7.26E-05 | -0.16 | 0.04 | 3.70E-05 |
| 5 | rs17730451 | *C5orf41* | 3’ flanking | A | 0.05 | 0.34 | 0.09 | 7.53E-05 | 0.33 | 0.09 | 1.35E-04 |
| 7 | rs849326 | *JAZF1* | upstream | C | 0.09 | 0.25 | 0.06 | 9.35E-05 | 0.24 | 0.06 | 1.07E-04 |

Tests of association at p≤1E-04 for Model 1 from single SNP linear regressions adjusted for study site and principal components (Model 1) and study site, principal components, year of birth, and BMI (Model 2) in 4,159 African American women from the PAGE Study are shown. For each significant test of association, the chromosome, rs number, nearest gene, location, coded allele, beta, standard error (SE), and p-value are given. Genes listed are nearest genes to the SNP as measured from the transcription start site for upstream SNPs or the transcription stop site for downstream SNPs. Abbreviations: CAF, coded allele frequency.
