# Supplementary material for: Genetic Variation and Reproductive Timing: African American Women from the Population Architecture Using Genomics and Epidemiology (PAGE) Study
Source: PLoS One. 2013 Feb 12;8(2):e55258. doi: 10.1371/journal.pone.0055258 (PMC3570525; doi:10.1371/journal.pone.0055258)
Supplement: Table S4 — Minor allele frequency comparisons of African American women in PAGE Study to HapMap CEU Panel. (DOCX) [file pone.0055258.s004.docx]

**Table S4: Minor allele frequency comparisons of African American women in PAGE Study to HapMap CEU Panel.**

| **SNP** | **Gene/Region** | **HapMap CEU AF (Allele)** | **PAGE Study AF (Allele)** |
| --- | --- | --- | --- |
| rs314277 | *LIN28B* | 0.13 (A) | 0.39 (A) |
| rs7759938 | *LIN28B* | 0.64 (T) | 0.46 (A) |
| rs4946651 | *LIN28B* | 0.48 (A) | 0.75 (A) |
| rs7861820 | 9q31 | 0.57 (T) | 0.11 (A) |
| rs4452860 | 9q31 | 0.72 (A) | 0.67 (A) |
| rs16991615 | *MCM8* | 0.09 (A) | 0.01 (A) |
| rs236114 | *MCM8* | 0.21 (A) | 0.09 (A) |
| rs7951733 | *FSHB* | 0.95 (A) | 0.99 (A) |
| rs769450 | *APOE* | 0.38 (A) | 0.38 (A) |
| rs7412 | *APOE* | 0.09 (T) | 0.10 (A) |
| rs1019731 | *IGF1* | 0.14 (T) | 0.02 (A) |
| rs9457827 | *IGF2R* | 0.05 (T) | 0.28 (A) |
| rs4135280 | *PPARG* | 0.98 (T) | 0.99 (A) |

Comparison of allele frequencies between PAGE Study African American women and HapMap CEU Panel. SNPs compared were previously associated with AM or ANM and directly genotyped on the Metabochip. Abbreviation: AF Allele Frequency.
